# A Pilot Study to Examine the Disparities in Water Quality between Predominantly Haitian Neighborhoods and Dominican Neighborhoods in Two Cities in the Dominican Republic

**DOI:** 10.3390/ijerph13010039

**Published:** 2015-12-22

**Authors:** Jessica Rogers-Brown, Ryan Johnson, Dominique Smith, Kim Ramsey-White

**Affiliations:** Center for Excellence on Health Disparities Research, School of Public Health, Georgia State University, Atlanta, GA 30302, USA; rjohnson115@student.gsu.edu (R.J.); dsmith174@student.gsu.edu (D.S.); kwhite@gsu.edu (K.R.-W.)

**Keywords:** health disparities, *E. coli*, water quality, waterborne disease, developing economies

## Abstract

Worldwide, diarrheal disease is a leading cause of death affecting over 1.7 million individuals annually. Much of this can be attributed to lack of clean water, sanitation and hygiene. Nearly all of these deaths occur in countries with developing economies. This public health problem is apparent in the island of Hispaniola; the island that is shared by Haiti and the Dominican Republic. Significant gaps in income between the countries have resulted in Haitians migrating into the Dominican Republic. While there has been increased migration into the Dominican Republic, many of the neighborhoods remain segregated. A cross-sectional analysis was conducted at 49 sites in the Dominican Republic. Samples were classified as being from a Haitian neighborhood or Dominican neighborhood and analyzed for microbial contamination. Overall, Haitian neighborhoods were found to have statistically significantly higher levels of contamination of both coliform and *E. coli*. The odds of having *E. coli* contaminated water in Haitian neighborhoods are 4.25 times as high as Dominican neighborhoods. The odds of having coliform contaminated water in Haitian neighborhoods are 4.78 times as high as Dominican neighborhoods. This study provides evidence of the disparity in access to clean drinking water for Haitian immigrants and highlights the need for further investigation.

## 1. Introduction

Lack of access to clean water is an ongoing global health crisis that disproportionally affects developing countries. Within these countries, there are often a subset of the population that is at a higher risk of contaminated water and waterborne pathogens. Worldwide, poor water quality, lack of sanitation and hygiene account for approximately 1.7 million deaths annually, with a large proportion of these deaths attributed to diarrhea [1]. In countries with developing economies, 9 out of 10 deaths are children [1].

Coliform and *E. coli* have long been a validated measure of fecal contamination in water. It is has also been established that improving water quality can also reduce diarrheal disease [2]. The presence of coliform and *E. coli* can lead to diarrheal disease and death, particularly in those of weakened immune systems, such as young children. The World Health Organization (WHO) estimated that contaminated water, in conjunction with poor hygiene and sanitation, accounts for 88% of diarrheal disease worldwide [3]. Additionally, in low-income settings, diarrheal disease is the leading cause of death among children ages 0–5 [4].

In the time span between 1990 and 2001, there has been a marked decrease in the availability of access to an improved drinking water source in the Dominican Republic [5].

Despite sharing one island, Haiti and the Dominican Republic have significant differences with regards to income equality. Haiti, which occupies the western portion of the island, is the poorest country in the western hemisphere. While residents of the Dominican Republic, although far from being rich, are still considered to be considerably better off than their Haitian neighbors [6]. This gap has long since been a catalyst for Haitian migration into the Dominican Republic, where Haitian citizens seek better employment options than what would be available in Haiti [7]. This began with the sugar plantations in the early 1900s and continues through today. The sugar plantations often recruited Haitians as migrant workers because the work was often viewed, by Dominicans, as being low-paying and beneath the dignity of many Dominican workers [8,9].

In September of 2013, the Constitutional Tribunal of the Dominican Republic ruled on an interpretation of the Dominican constitution that retroactively revoked Dominican citizenship to any children born to irregular migrants from 1929 forward. This ruling impacted over 200,000 individuals of Haitian descent living in the Dominican Republic. In 2005 the case of *Yean and Bosico v. Dominican Republic* resulted in a constitutional interpretation of this ruling that was determined to be discriminatory and a violation of human rights by the Inter-American Court of Human Rights (IACHR) [10]. Unfortunately, this arbitrary and discriminatory violation of international law only adds to the stigma experienced by Haitian immigrants in the Dominican Republic.

Previous research has shown that, in the Dominican Republic, predominantly Haitian neighborhoods bear a disproportionate burden of disease. According to a 2007 study by the National Statistics Office, in the Dominican Republic, diarrheal disease occurs at much higher rates in the Haitian batayes (slum areas that were once designed to house sugar cane workers) than in the country in general. In a given two-week period, 21% of children in the Haitian batayes contracted diarrheal disease—while the rate in the country as a whole was only 14%. In the same study, the infant mortality rate was 36/1000 in the country overall while in the batayes that number was 44/1000 [11]. Since the residents of Haitian neighborhoods bear a disproportionate burden of diarrheal disease, it is important to understand if water quality is a factor.

This study was designed to assess if there are differences in water contamination, by coliform and *E. coli*, between Dominican neighborhoods and Haitian neighborhoods in two areas of the Dominican Republic.

## 2. Experimental Section

### 2.1. Neighborhood Data

Neighborhood type was determined by multiple key informant data. Key informants were asked about the make-up of the neighborhood (Haitian or Dominican). Key informants were also asked to classify the neighborhoods into socio-economic quadrants. Data was collected on two key informants per city and compared. At sample collection the language spoken at the site (or closest to the site for community taps) were used as a confirmation tool for neighborhood type. Socio-economic quadrants were used to ensure that socio-economic status was evenly distributed in both neighborhoods and therefore not a confounding factor.

### 2.2. Environmental Data

A cross-sectional study design was used and samples were collected based on a convenience sampling method. The samples were selected from the two pre-identified cities during the months of May–June 2014. Data collectors walked through the neighborhoods in these cities collecting samples with the criteria that the samples be at least two blocks from each other. Sites were selected from within that criteria based on physical and permissible access. Forty-nine samples were collected in Santo Domingo and Sousa, Dominican Republic. The inclusion criteria were water that was used for cooking, food washing, or drinking: all samples that could be ingested during normal daily activities. One sample was excluded from analysis because there was not enough information collected to categorize the sample. The total number of samples used for analysis was 48.

Samples were collected from sources that were publicly accessible. This included source water, tap water, household stored water that had been purchased from a water dealer, household stored water that had been collected from a community water tap, water from a community tap, purchased ice, purchased water from small bottles, purchased water from five gallon containers, purchased water from a restaurant, and water from business taps. The water samples were categorized as tap water (*n* = 31), stored water (*n* = 9), or source water (directly from water source e.g., river) (*n* = 8).

Samples were collected aseptically with U.S. Environmental Protection Agency (EPA) compliant 100 mL sampling bottles. Upon collection, samples were measured for temperature and Global Positioning System (GPS) coordinates were recorded. Samples were then packed on ice until they could be placed in refrigeration (within six hours maximum).

Samples were analyzed using ColiScan EasyGel manufactured by Micrology Laboratories. Five milliliters of water was added to an inoculum using sterile milliliter droppers. The inoculum and water were swirled and placed on the prepared Petri dish. Then the samples were incubated at high room temperature (between 24.4 and 32.2 degrees Celsius) and monitored every twelve hours. Colony counts were collected between 36–48 h.

Colonies were counted by total coliforms and *E. coli* colonies. All samples above 224 colonies of either category were considered ‘too numerous to count’. Photographic evidence of each plate was collected in the field. Samples were later re-counted using a 40-inch zoom enabled screen. There were four samples that had discrepancies in the number. In those cases the lowest number of colonies was used. Data was analyzed using SAS 9.2.

## 3. Results and Discussion

The unit of analysis was water sample. Since the total coliform and *E. coli* samples were not normally distributed Logirithic10 transformations were used to better understand the distribution, but the samples were still not normally distributed, with peaks at 0 and peaks at ‘too numerous to count’. The data were categorized into presence and absence of coliform and presence or absence of *E. coli*.

Both Haitian neighborhood and Dominican neighborhoods tested positive for the presence of coliform. Dominican neighborhoods showed coliform contamination in 59% of the samples while Haitian neighborhoods had coliform contamination in 88% of the samples. Neighborhood differences in coliform contamination were shown to be statistically significant using Fisher’s exact test (*p* = 0.05; FET). Odds ratio analysis showed that the odds of having coliform contaminated water, for a Haitian neighborhood, is 4.78 times the odds of having coliform contaminated water for a Dominican neighborhood (see Table 1).

**Table 1:** Presence of coliform bacteria in water samples.

Coliform Bacteria	Neighborhood Type	
	Dominican	Haitian
Coliforms Absent	13	2
Coliforms Present	19	14

*E. coli* was also present in both neighborhoods categories. *E. coli* was present in 28% of the Dominican neighborhood samples. *E. coli* was present in 63% of the Haitian neighborhood samples. A statistical comparison was made using a Chi-squared analysis. The analysis showed a statistical difference between the two groups *Χ*^2^ (1, *N* = 48) = 5.27, *p* = 0.0217. The odds ratio analysis showed that the odds of having *E. coli* contaminated water for a Haitian neighborhood is 4.25 times the odds of having *E. coli* contaminated water when compared to a Dominican neighborhood (see Table 2).

**Table 2:** Presence of *E. coli* bacteria in water samples.

*E. coli* Bacteria	Neighborhood Type	
	Dominican	Haitian
*E. coli* Absent	23	6
*E. coli* Present	9	10

The samples were also examined by source of water to detect if stored water was a possible confounder of the true relationship between neighborhood type and presence of contamination. In a chi-squared analysis there was not a significant association between source of water and presence of contamination.

A comparison was also made between these samples and the World Health Organization (WHO) water quality guidelines. These guidelines dictate that any water is designated as very high risk when *E. coli* most probable number (MPN) is greater than 100/100 mL; a high risk when *E. coli* MPN ranges from greater than 10 to 100/100 mL; as intermediate risk when the *E. coli* MPN is between 1 and 10/100 mL; and, low risk when the *E. coli* MPN is less than 1/100 mL [11]. Based on these guidelines, more than 40% of the samples overall were at least immediate risk. When examining the risk categorization from the point of view of the neighborhoods, we found a statistically significant difference between Haitian neighborhoods and Dominican neighborhoods. Dominican neighborhoods had a classification of at least intermediate risk in 28.13% of the samples. Haitian neighborhoods experience intermediate risk in 62.5% of samples, more than double the rate of Dominican neighborhoods (FET, *p* = 0.0315).

## 4. Conclusions

Overall the results show a disproportionately higher level of water contamination in Haitian neighborhoods than in Dominican neighborhoods. The hypothesis that Haitian neighborhoods are at a higher risk for water contamination is supported by the data. This study confirms the results of microbial contamination reported in previous studies [11]. Furthermore, this project provides evidence that, not only are there high levels of contaminated water samples, but that there is a disparity in the access to uncontaminated water. There is still a need to identify the mediating factors in the relationship between neighborhood type and water quality.

This study provides evidence that there is a significant difference in the water being used in Haitian and Dominican households. These findings highlight a potential cause for the disparities in health, particularly the occurrence of diarrheal disease. Higher levels of water contamination, as evidenced by this paper, provide a strong possible explanation for the health disparities found in previous research [12].

### 4.1. Limitations

The study was a pilot study to determine if more extensive research is warranted. As such, the sample size was small and due to the small sampling size was small there were not enough data points to complete regression analysis to model any other potential confounders. The data calls for a larger scale study that would allow for more rigorous data analysis.

Because of the sampling administration, the coverage was limited to two urban geographic areas of the Dominican Republic. Any future studies should seek to increase the geographic area of the study to assess if the same results would also be found in more rural areas of the country.

### 4.2. Future Directions

Future research is certainly warranted in the area of water quality studies in the Dominican Republic. Based on these results, future research should seek to expand the geographic area studied to better understand the results in the context of the entire country. Future research should attempt to pinpoint the sources of contamination. Additionally, the sheer extent of contamination warrants interventional research. Interventions should be translated in both Spanish and Creole to ensure the broadest width of coverage. If public health promotional materials are limited to Creole only, the problem may be interpreted as a “Haitian-problem”, and therefore only increase the current stigma and disparity plaguing the country.

More research is needed to assess the scope of the problem. Once the scope is established interventional research could have a huge impact on myriad areas of health and well-being issues. Chung *et al.*, were able to show that by manipulating water supply as a variable, there would be a statistically significant improvement in poverty and hunger, gender equality, child mortality, maternal health, and diseases [13]. Future research is essential to assess the scope of water contamination in Haitian neighborhoods in order to instigate targeted interventions to improve the overall health of those living in these communities.
